# Deep learning for automatic detection of hepatocellular carcinoma in dynamic contrast-enhanced MRI

**DOI:** 10.1007/s00261-025-05249-4

**Published:** 2025-11-11

**Authors:** Killian Monnin, Patrick Jeltsch, Lucia Fernandes-Mendes, Vasco Cazzagon, Marianna Gulizia, Mario Jreige, Montserrat Fraga Christinet, Raphael Girardet, Clarisse Dromain, Jonas Richiardi, Naik Vietti-Violi

**Affiliations:** 1 University of Lausanne, Lausanne, Switzerland; 2 Department of Radiology, Lausanne University Hospital, Lausanne, Switzerland; 3 Department of Gastro-enterology, Lausanne University Hospital, Lausanne, Switzerland; 4 Department of Radiology, South Metropolitan Health Service, Murdoch, Australia

**Keywords:** Liver, HCC, MRI, Machine learning, Lesion detection

## Abstract

**Objective:**

Develop a deep learning model for automatic hepatocellular carcinoma (HCC) detection in T1 weighted imaging (WI) Dynamic Contrast-Enhanced (DCE) liver MRI using extracellular contrast agent, and to analyze its performance at both patient and lesion levels.

**Materials and methods:**

This retrospective study included two cohorts, the first included patients (*N* = 296) undergoing HCC surveillance with diagnosed HCC as well as negative cases. The 233 HCC negative patients and 12 HCC positive patients were used to create the HCC Surveillance test set, aiming to evaluate patient level performance on simulated screening conditions. The second included Pre-Ablation patients (*N* = 67), all positive for HCC, used as test set for lesion level evaluation and to measure generalization performance. The two largest public liver lesion datasets (CT, *N* = 1037 and MRI, *N* = 485) were used for pre-training the algorithms. An attention U-Net model was trained to segment and detect HCC and was compared to the state-of-art nnU-Netv2. Diagnostic accuracy was evaluated using sensitivity, specificity, mean false positives per patient, PPV and NPV, the Area Under the Curve (AUC) of the Free-Response Operating Characteristic (FROC) curves and the Receiver Operating Characteristic (ROC) curves.

**Results:**

The final population included 363 patients (58 ± 11 years; 284 men; 247 lesions): 51 HCC positive patients (113 lesions) used in training set, 245 patients (12 HCC positive with 21 lesions, 233 HCC negative) in the HCC Surveillance testing set, 67 HCC positive patients (113 lesions) in the HCC Pre-Ablation testing set. At patient level, 83% sensitivity and 72% specificity [AUC of 0.80 (95% CI: 0.66–0.91)] was measured on the HCC Surveillance test set. At lesion level, 80% of sensitivity for a mean false positive per patient of 1 was measured on the HCC Pre-Ablation test set with the pre-trained model with a FROC AUC of 0.82 (95% CI: 0.77–0.88), significantly outperforming the nnU-Netv2 at 0.61 (95% CI: 0.52–0.69, *p* < 0.01).

**Conclusions:**

Both patient-level and lesion-level achieved 80% HCC detection sensitivity by using a deep learning segmentation neural network pre-trained from large open datasets. This performance highlights the translational potential of such tools in the clinical workup of patients at risk of HCC.

**Supplementary Information:**

The online version contains supplementary material available at 10.1007/s00261-025-05249-4.

## Introduction

Hepatocellular carcinoma (HCC) is an important cause of death worldwide and is predicted to rise in the forthcoming years [1]. Early identification of the tumor is crucial to increase the prospects of efficacious treatment [2]. Currently, MRI is performed in case of anomaly detected with ultrasound (US) in order to confirm HCC diagnosis, using the Liver Imaging Reporting and Data System (Li-RADS) v2018 [3]. Li-RADS is mainly based on lesion appearance across multiphasic contrast-enhanced sequences and allows to diagnose HCC on Dynamic Contrast-Enhanced (DCE) imaging without the need for biopsy in conditions of chronic liver disease.

US is known for a limited sensitivity for HCC detection, particularly in case of large body habitus or cirrhosis with a sensitivity of 47% [2]. MRI offers superior sensitivity for HCC detection (84–86%) but it is not used currently for HCC screening due to its higher costs, long scan times and limited availability [4, 5]. An alternative could be abbreviated MRI (AMRI) [5], reducing cost and scan times while keeping high sensitivity for HCC detection. HCC detection with AMRI could be optimized using a deep learning-based computer-aided decision tool, improving lesion detection rate, reducing radiologists’ interpretation times and increasing reproducibility.

The task of automated HCC detection in MRI can be addressed with various methods (such as machine learning for classification or object detection, or radiomics approaches) but automatic segmentation remains the preferred approach due to its ability to provide precise localization and detailed lesion boundaries essential for characterizing imaging features used in LI-RADS categorization (e.g., size, arterial enhancement, washout, enhancing capsule). In a screening or diagnostic setting, automated segmentation can serve as a foundation for downstream applications such as lesion classification or treatment planning.

HCC detection by segmentation in DCE sequences with extracellular contrast was approached by Bousabarah et al., feeding a 3D U-Net model with concatenated native, arterial, venous and delayed phases [6]. Although this method provided satisfactory HCC detection (75% lesion-level sensitivity and specificity), the performance evaluation was limited to a few lesions (*n* = 34). Another group proposed to detect HCC in dynamic abbreviated MRI (T2WI + T1WI DCE performed with liver specific contrast agent) with the 2D DINOv2 architecture and reached a lesion-level recall of 91% [7]. Similarly, a recent study trained a U-Net model with DCE MRI with extracellular contrast agent and obtained a sensitivity of 96% and 97% at lesion level and 89% and 94% at patient level on internal (91 lesions) and external (62 lesions) test sets [8]. However, no HCC negative patients and no hepatic lesions other than HCC were included to evaluate the performance of these algorithms in terms of false positives, which limits their evaluation for real-world applicability. This former study was performed in China where viral hepatitis is predominant in the HCC screening population, completely different from the population of HCC screening in the western countries (mainly composed of patients with metabolic dysfunction-associated steatohepatitis and alcohol abuse) [9].

The aim of our study is to develop a deep learning algorithm for automatic HCC segmentation and detection in T1WI DCE sequences with extracellular contrast agent. We investigated the HCC detection in a western population where alcohol-related liver disease is the predominant predisposing factor. Therefore, these data include cases with clinical evidence of advanced cirrhosis (Child-Pugh score B or C) characterized by the presence of fibrosis, increasing the complexity of the task of HCC detection [10, 11].

## Materials and methods

### Study design and cohorts

This study included retrospective unicentric data acquired in a hospital and from two different cohorts of patients at high risk of HCC. In addition, data from two public datasets were used as pre-training. The study was conducted in accordance with the TRIPOD + AI reporting guidelines [12].

#### HCC Surveillance cohort

**Fig. 1:**
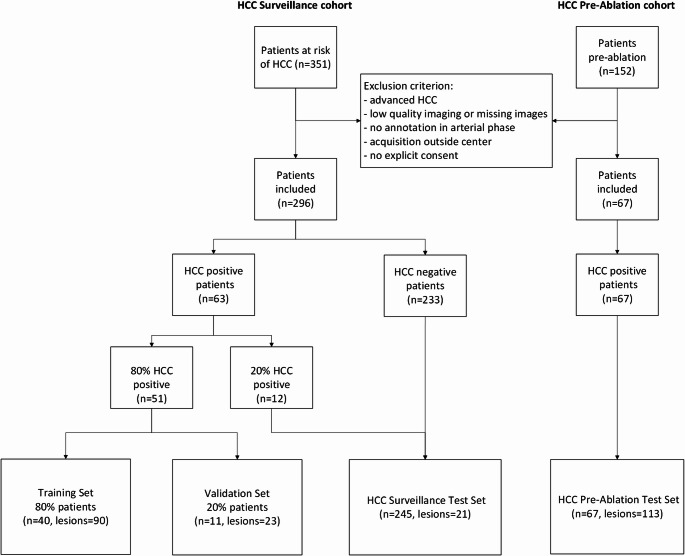
Flow chart of patient selection

The HCC Surveillance cohort is a retrospective study which was approved by the local institutional review board including 351 patients with chronic liver disease with extracellular contrast liver MRI [13]. This study included the MRI of 296 patients (63/82 HCC positive and 233/269 HCC negative) after patient exclusion: patients with advanced HCC (*n* = 15), no lesion annotation on arterial phase (*n* = 2), bad quality or missing images (*n* = 2) and missing of explicit consent (*n* = 36) (Fig. 1). The HCC positive patients of the Surveillance dataset were split into random 5-folds cross-validation sets (51 HCC positive patients, 113 lesions) and a test set (12 HCC positive patients, 21 lesions, 233 HCC negative patients). That test set was used to evaluate patient level performance with a realistic HCC screening prevalence (5%).

#### HCC Pre-Ablation cohort

The second cohort is the HCC Pre-Ablation cohort, a retrospective analysis of a prospective study approved by local institutional review [14], which included 152 patients with chronic liver disease with extracellular contrast liver MRI before HCC percutaneous ablation. A total of 67 patients (all HCC positive) were included in the present study after exclusion of patients outside the center (*n* = 85) (Fig. 1). That cohort was used to create the Pre-Ablation test set (67 patients, 113 lesions), to evaluate lesion level performance.

#### External pre-training datasets

Deep neural networks have many trainable parameters and generally perform better with more data. Pre-training followed by fine-tuning refers to using data related to the task of interest (HCC lesion segmentation) to train the neural network, then fine-tuning (retraining slightly) on the final data, as previously suggested [15]. Two different publicly available datasets were used for pre-training in the present study.

- The Liver Tumor Segmentation (LiTS) dataset includes CT from 131 patients for a total of 1037 lesions. The dataset includes HCC lesions but also cholangiocarcinoma and metastatic liver lesions [16]. That dataset was used to pre-train the model with general knowledge of liver anatomy and lesion localization on CT imaging.
- The public Liver Lesion Diagnosis on Multi-Phasic MRI (LLD-MMRI) dataset includes 500 patients each with annotated liver lesions in multi-phasic MRI. That dataset was used to further adapt the model to the same imaging modality (MRI) as our study but with a different population (China) and different lesion types (cysts, hemangiomas, focal nodular hyperplasia, abscesses, cholangiocarcinoma, metastasis and HCC) [17].

### Image parameters and analysis

All MRI were standard clinical liver MRI following the LI-RADS technical recommendations. Histopathology confirmation was used when available and in other cases, diagnosis was based on imaging criteria according to LI-RADS v2018 [3]. For the present study, the following sequences were included: axial T1WI pre-contrast, arterial, portal venous and delayed phases after extracellular contrast injection (gadoteric acid). 3D liver annotations were performed independently by a radiology technician. Lesion segmentations were performed independently on all sequences by a radiologist in training using all patient information including pathology and follow-up imaging as reference. All segmentations were performed using Mint ^TM^ Software and were reviewed by an expert abdominal radiologist (8 years of experience) who also reviewed all lesions and scored them using LI-RADS [3]. While HCC positive patients all have at least one confirmed HCC, some non-HCC lesions were also included in the analysis.

The HCC Surveillance dataset was composed of 96% of 3T and was collected with 10 different MRI scanners from 3 manufacturers: Siemens, GE and Philips. The HCC Pre-Ablation dataset of 45% of 3T was acquired with 14 MRI scanners from the same manufacturers. More details on acquisition protocols and MRI parameters are available in Supplementary material 1.

### Neural network segmentation models

**Fig. 2:**
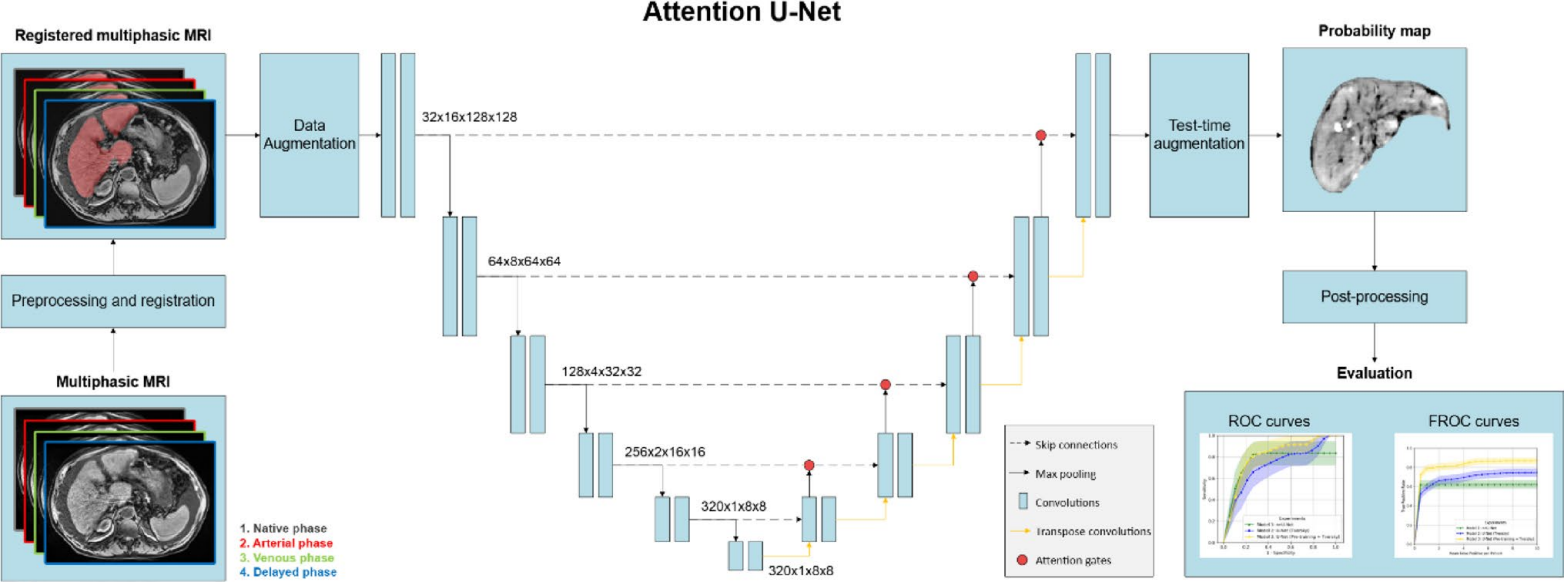
Workflow of the proposed method. Additional information on the attention gate principle is described in Supplementary Material 2

The methodology of HCC detection consisted in training U-Net-based architectures to segment and localize HCC lesions, as illustrated in Fig. 2. The multiphasic MRI sequences were first registered using the liver mask generated by a pre-trained nnU-Net. The liver region was extracted from the multiphasic images and used as input for training lesion segmentation models restricted to the liver region. We trained a baseline nnU-Net for this task to compare its performance with two other methods designed to prioritize sensitivity or specificity.

#### nnU-Net

We selected the most recent version of the 3D full-resolution architecture nnU-Netv2 [21], a competitive network in many medical imaging competitions, as a benchmark network to compare our models.

#### U-Net with Tversky loss

The model architecture was the Attention U-Net [23] implemented in the MONAI library. The model was trained with the Tversky loss function (α = 0.1 and β = 0.9) to penalize false positive predictions less, and false negatives more, creating a sensitive model.

#### U-Net Pre-trained + Tversky loss

The second model was the same architecture, but pre-trained from scratch on the LiTS dataset [16] (489 patients), fine-tuned on LLD-MMRI dataset [17] (131 patients) and further fine-tuned with the HCC Surveillance cohort. In addition, in the loss function, 10 times greater penalization was set on HCC lesions to focus the model on HCC recognition. The Tversky loss was used with α = 1.0 and β = 0.9 to reduce false positive detections, creating a specific model.

More details about the neural networks training configuration, pre-processing and post-processing techniques used on prediction maps are described in Supplementary material 2.

### Metrics and statistical analysis

The primary metrics used for lesion level detection evaluation were the sensitivity, the mean number of false positive per patient (average FPPI [26] with image defined here as the 3D patient volume) and the area under the curve (AUC) of the free-response operating characteristic (FROC) curves [27]. At patient level, the performance was evaluated using sensitivity, specificity, the AUC of the receiver operating characteristics (ROC) curve, the positive and negative predictive value (PPV and NPV), along with prevalence-adjusted PPV/NPV calculated for a 5% prevalence to enable fair comparison with other studies. The 95% confidence intervals of the AUC were computed using a non-parametric bootstrap approach. We performed *N* = 1000 bootstrap resamples, drawing patients with replacement, and computed the AUC for each resample. We employed permutation tests to test for differences between neural networks. For descriptive statistics, Fisher and Mann-Whitney U tests were used with the SciPy Python library v1.9.1 to assess test dataset differences (Supplementary material 3).

## Results

### Patient population

The final study population included 363 patients (58 ± 11 years, 284 men) with 130 HCC positive patients (247 lesions) and 233 HCC negative patients. The HCC Surveillance cohort included a total of 296 patients with 63 HCC positive patients (including 134 lesions) and 233 HCC negative patients. The HCC positive patients of the Surveillance dataset were split into random 5-folds cross-validation sets (51 HCC positive patients, 113 lesions) and a test set (12 HCC positive patients, 21 lesions). Patient demographics are described in Table 1.

**Table 1:** Patients characteristics by training, validation and testing data splits

	Surveillance cohort		Pre-Ablation cohort	
	Training and Validation Set	Test Set	Test Set	*p-value*
Number of patients	51	245	67	
Mean age	62.0 ± 8.0	56.0 ± 12.0	64.0 ± 9.0	*< 0.001*
Sex				*0.168*
Female	16 (31.4%)	54 (22.0%)	9 (13.4%)	
Male	35 (68.6%)	191 (78.0%)	58 (86.6%)	
Ethnicity				*0.030*
African	3 (5.9%)	17 (6.9%)	3 (4.5%)	
Asian	5 (9.8%)	17 (6.9%)	0 (0.0%)	
Caucasian	42 (82.4%)	204 (83.3%)	60 (89.6%)	
South America	1 (2.0%)	4 (1.6%)	0 (0.0%)	
Other	0 (0.0%)	3 (1.2%)	4 (6.0%)	
Liver disease etiology				*< 0.01*
HCV	24 (47.1%)	58 (23.7%)	21 (31.3%)	
HBV	4 (7.8%)	44 (18.0%)	11 (16.4%)	
Alcohol consumption	28 (54.9%)	120 (49.0%)	50 (74.6%)	
Metabolic disease	0 (0.0%)	2 (0.8%)	21 (31.3%)	
Cirrhosis				*< 0.01*
No	1 (2.0%)	35 (14.3%)	1 (1.5%)	
Yes	50 (98.0%)	208 (84.9%)	66 (98.5%)	
Child-Pugh class				*0.025*
A	34 (68.0%)	159 (77.6%)	50 (74.6%)	
B	15 (30.0%)	33 (16.1%)	17 (25.4%)	
C	1 (2.0%)	13 (6.3%)	0 (0.0%)	

Variables are presented as count (%) and test sets were compared using the Fisher test for categorical variables and the Mann-Whitney U test for continuous variables

*HCV* Hepatitis C Virus, *HBV* Hepatitis B Virus, *HCC* hepatocellular carcinoma

Among the 247 lesions included in the present study, 201 were confirmed HCC and 46 were not HCC (Table 2). Among HCC lesions 176 were classified LR-5, 17 biopsy-proven LR-4, 1 biopsy-proven LR-3, 2 biopsy-proven LR-M and 5 LR-TIV. Among non-HCC lesions, 45 were presumed or biopsy-proven dysplastic nodules (18 LR-4, 27 LR-3) and 1 was a cholangiocarcinoma (1 LR-M). The HCC negative patients included non-HCC lesions, which were not annotated.

**Table 2:** Lesion characteristics

Data split	Surveillance cohort		Pre-Ablation cohort	
	Training and Validation Set	Test Set	Test Set	*p-value*
Patients				
HCC positive	51	12 (4.9%)	67	
HCC negative	–	233 (95.1%)	–	
Number of lesions	113	21	113	
Mean lesions per patient	2.14 ± 1.28	1.75 ± 0.83	1.77 ± 1.18	*0.67*
Mean lesion diameter (mm)	21.1 ± 11.5	23.5 ± 16.7	28 ± 18.7	*0.12*
Confirmed HCC lesions	81	16	104	
Mean lesion diameter (mm)	24.77 ± 12.62	29.6 ± 18.26	29.87 ± 19.25	
LR-5	71 (87.7%)	14 (87.5%)	91(87.5%)	
LR-4	5 (6.2%)	–	12(11.5%)	
LR-3	1 (1.2%)	–	–	
LR-M	1 (1.2%)	–	1(1%)	
LR-TIV	3 (3.7%)	2 (12.5%)	–	
Non-HCC	32	5	9	
Mean lesion diameter (mm)	15.54 ± 4.68	10.28 ± 1.21	14.13 ± 4.63	
LR-5	–	–	–	
LR-4	15 (46.9%)	3 (60%)	–	
LR-3	17 (53.1%)	2 (40%)	8 (88.9%)	
LR-M	–	–	1 (11.1%)	
LR-TIV	–	–	–	

Variables are presented as count (%) and test sets compared using the Fisher test for categorical variables and the Mann-Whitney U test for continuous variables

*HCC* hepatocellular carcinoma

### Patient level detection

**Fig. 3:**
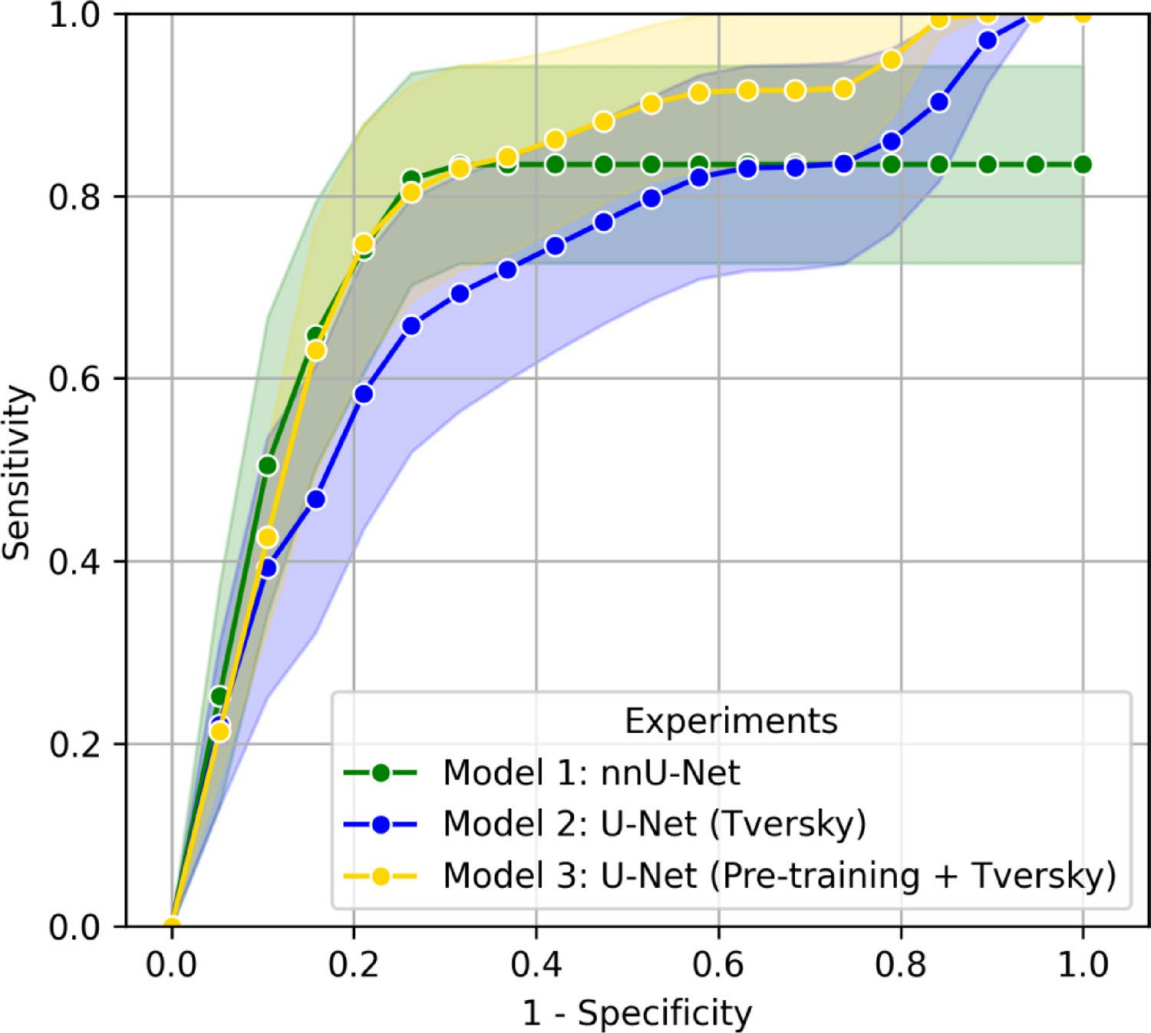
Patient level ROC Curves were calculated to measure the performance of each model on the HCC Surveillance test set. The mean and the standard deviation are represented as shaded regions around each curve. *ROC* receiver operating characteristics. *U-Net (Tversky)* U-Net trained from scratch with Tversky loss, *U-Net (Pre-training + Tversky)* U-Net pre-trained on LiTS and sequentially fine-tuned on LLD-MMRI and the HCC Surveillance test set

The detection performance of the models at patient level is illustrated in Fig. 3 and was evaluated with the HCC Surveillance test set. The nnU-Net achieved an AUC of 0.80 (95% CI: 0.67–0.92) and the U-Net (Tversky) an AUC of 0.72 (0.53–0.87). The U-Net (Pre-training + Tversky) achieved a similar performance as the nnU-Net at patient level with an AUC of 0.80 (0.66–0.91).

### Lesion level detection

**Fig. 4:**
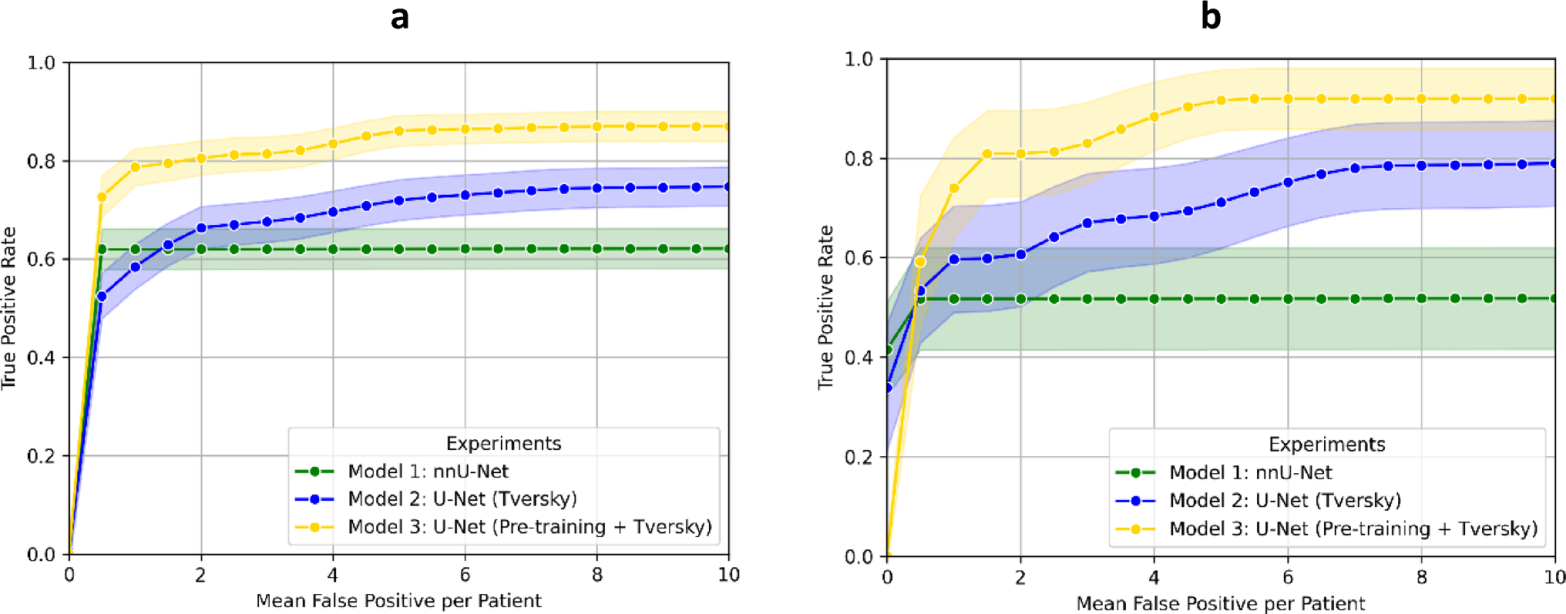
Free receiver operating characteristics curves on HCC Surveillance test set (**a**) and HCC Pre-Ablation test set (**b**). The mean and the standard deviation are represented as shaded areas around the curves. *U-Net (Tversky)* U-Net trained from scratch with Tversky loss, *U-Net (Pre-training + Tversky)* U-Net pre-trained on LiTS and sequentially fine-tuned on LLD-MMRI and the HCC Surveillance test set

The detection performance of the models to detect confirmed HCC lesions was evaluated on each test set (Fig. 4). In the HCC Surveillance test set, the U-Net (Pre-training + Tversky) demonstrated the highest performance [AUC = 0.82 (95% CI: 0.72–0.96, *p* < 0.05)] compared to the nnU-Net [AUC = 0.55 (0.31–0.80)] and the U-Net (Tversky) [AUC = 0.68 (0.53–0.89)]. In the HCC Pre-Ablation test set, the U-Net (Pre-training + Tversky) also demonstrated the highest performance [AUC = 0.82 (0.77–0.88, *p* < 0.01)] compared to the nnU-Net [AUC = 0.61 (0.52–0.69)] and the U-Net (Tversky) [AUC = 0.69 (0.61–0.76)].

The performance was stratified at lesion level by different Li-RADS categories to analyze the detection performance of the models (Table 3). The U-Net (Pre-training + Tversky) demonstrated the highest AUC for the detection of HCC lesions and LR-5 in both datasets (*p* < 0.05 in the HCC Surveillance test set and *p* < 0.01 in the HCC Pre-Ablation test set). The U-Net (Tversky) performed better than the nnU-Net in the testing sets to detect HCC, LR-5 and LR-4 lesions and detected fewer LR-3. Training and validation results are presented in Supplementary material 4.

**Table 3:** FROC curves AUC

HCC Surveillance Test Set					
	All lesions (*n* = 21)	HCC (*n* = 16)	LR-5 (*n* = 14)	LR-4 (*n* = 3)	LR-3 (*n* = 2)
nnU-Net	0.51	0.55	0.49	0.32	**0.49**
U-Net (Tversky)	*0.63*	*0.68*	*0.63*	*0.83*	0.00
U-Net (Pre-training + Tversky)	0.79*	0.82*	0.80*	**0.91**	*0.38*

HCC Pre-Ablation Test Set					
	All lesions (*n* = 113)	HCC (*n* = 104)	LR-5 (*n* = 91)	LR-4 (*n* = 12)	LR-3 (*n* = 10)
nnU-Net	0.61	0.61	0.65	0.24	*0.68*
U-Net (Tversky)	*0.67*	*0.69*	*0.72*	*0.43*	0.58
U-Net (Pre-training + Tversky)	0.81**	0.82**	0.86**	**0.58**	**0.73**

*AUC* area under the curve, *U-Net (Tversky)* U-Net trained from scratch with Tversky loss, U-Net (Pre-training + Tversky): U-Net pre-trained on LiTS and sequentially fine-tuned on LLD-MMRI and the HCC Surveillance test set

*: *p-value < 0.05*; **: *p-value < 0.01*. p-values calculated from permutation test between the nnU-Net and the U-Net (Pre-training + Tversky)

The highest value for each column is shown in bold and the second highest in italics

A comparison of the model performance obtained for HCC detection is presented in Table 4. Overall, the U-Net (Pre-training + Tversky) showed to be the best trade-off for high sensitivity at lesion level (81%) and patient level (83%) and high specificity at patient level (72%) in the HCC Surveillance test set. The PPV of the U-Net (Pre-training + Tversky) was 13.3%, 14.5% for the nnU-Net and 11.9% for the U-Net (Tversky) at patient level. The NPV was similar between the U-Net (Pre-training + Tversky) and the nnU-Net (98%) and slightly lower (97.7%) for the U-Net (Tversky). In the HCC Pre-Ablation test set, the U-Net (Pre-training + Tversky) achieved a sensitivity of 80% with a mean false positive rate of 0.89. The model evaluation with different intersection over union (IoU) thresholds is available in Supplementary material 5.

**Table 4:** Detection metrics

	Lesion level		Patient level			
	Sensitivity	Mean FP per patient	Sensitivity	Specificity	PPV	NPV
HCC Surveillance Test Set						
nnU-Net	56%	**0.27**	*75%*	**77%**	**14.5%**	*98.4%*
U-Net (Tversky)	*62%*	*2.5*	67%	*74%*	11.9%	97.7%
U-Net (Pre-training + Tversky)	**81%**	2.68	**83%**	72%	*13.3%*	**98.8%**
HCC Pre-Ablation Test Set						
nnU-Net	*63%*	**0.45**				
U-Net (Tversky)	59%	0.92				
U-Net (Pre-training + Tversky)	**80%**	*0.89*				

U-Net (Tversky): U-Net trained from scratch with Tversky loss, U-Net (Pre-training + Tversky): U-Net pre-trained on LiTS and sequentially fine-tuned on LLD-MMRI and the HCC Surveillance test set

*PPV* positive predictive value, *NPV* negative predictive value

The highest value for each column is shown in bold and the second highest in italics

### True positives and true negatives

**Fig. 5:**
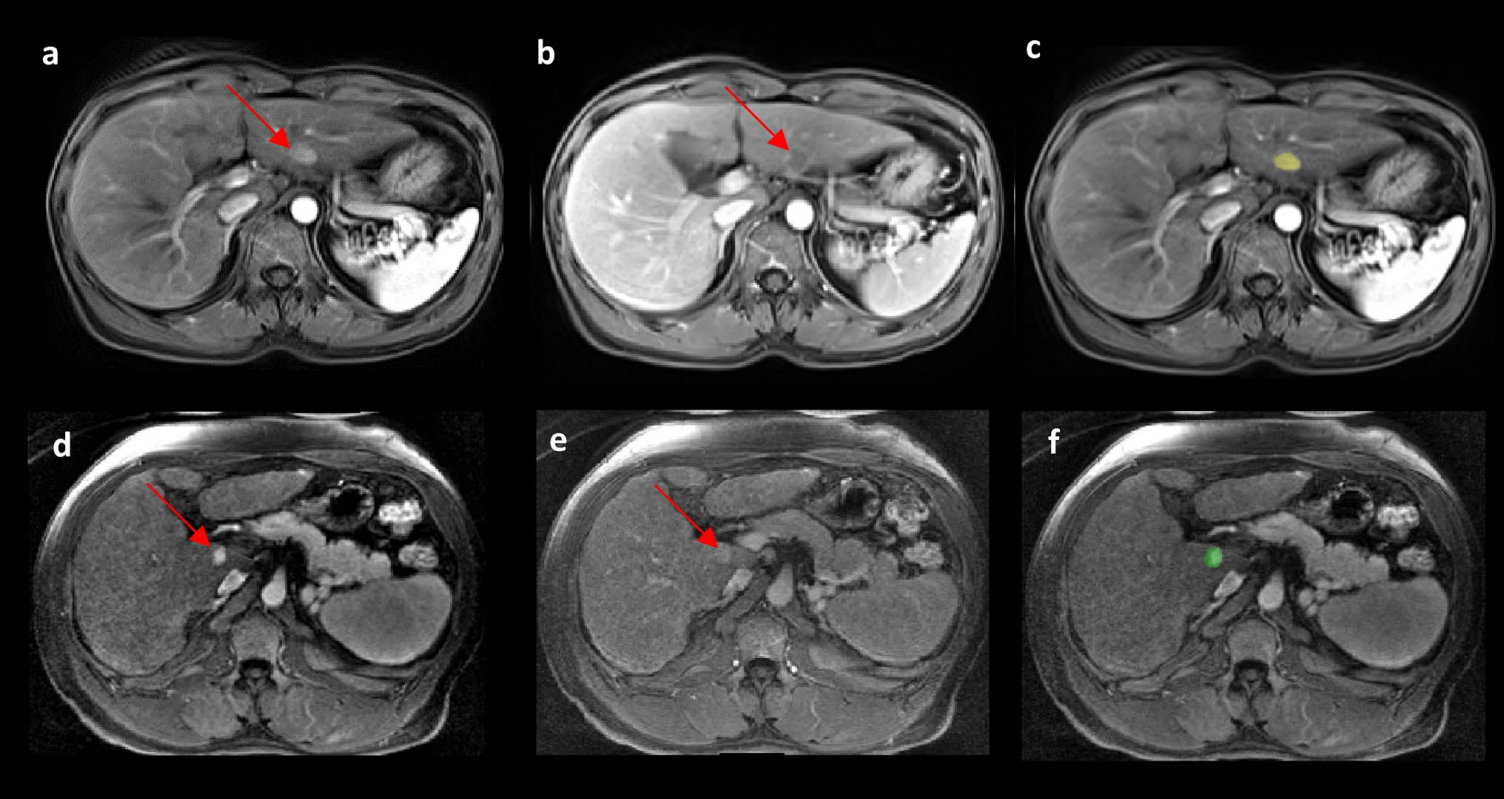
Example of true positive and true negative predictions of the U-Net (Pre-training + Tversky) from the HCC Pre-Ablation test set. In the first column on the left **a** and **d** are the arterial phases, in the second **b** and **e** are the venous phases and in the last the predictions or the ground truth (**c** and **f**). The tumor model predictions are highlighted in yellow (**c**) and the tumor ground truth in green (**f**). **a–c** True positive prediction of an HCC lesion (arrow) on arterial and venous phases (**a** and **b**) with corresponding model segmentation (**c**). **d–f** Example of a true negative prediction on a non-HCC (arrow) lesion with arterial phase hyperenhancement (**d**, arrow) and without washout or enhancing capsule on venous phase (**e**). Its segmentation mask **f** that was correctly not detected by the model. Full multiphasic visualizations are presented in Supplementary material 6. *HCC* hepatocellular carcinoma. *U-Net (Pre-training + Tversky)* U-Net pre-trained on LiTS and sequentially fine-tuned on LLD-MMRI and the HCC Surveillance test set

At 83% sensitivity and 72% specificity at patient level, 10/16 HCC lesions were detected and 5/5 of non-HCC lesions present in the HCC Surveillance test set were correctly not detected. At a sensitivity of 80% and a mean of 1 false positive per patient, 83/104 HCC lesions were detected and 6/9 of LR-3 lesions were detected in the HCC Pre-Ablation test set (Fig. 5).

### False positives and false negatives

**Fig. 6:**
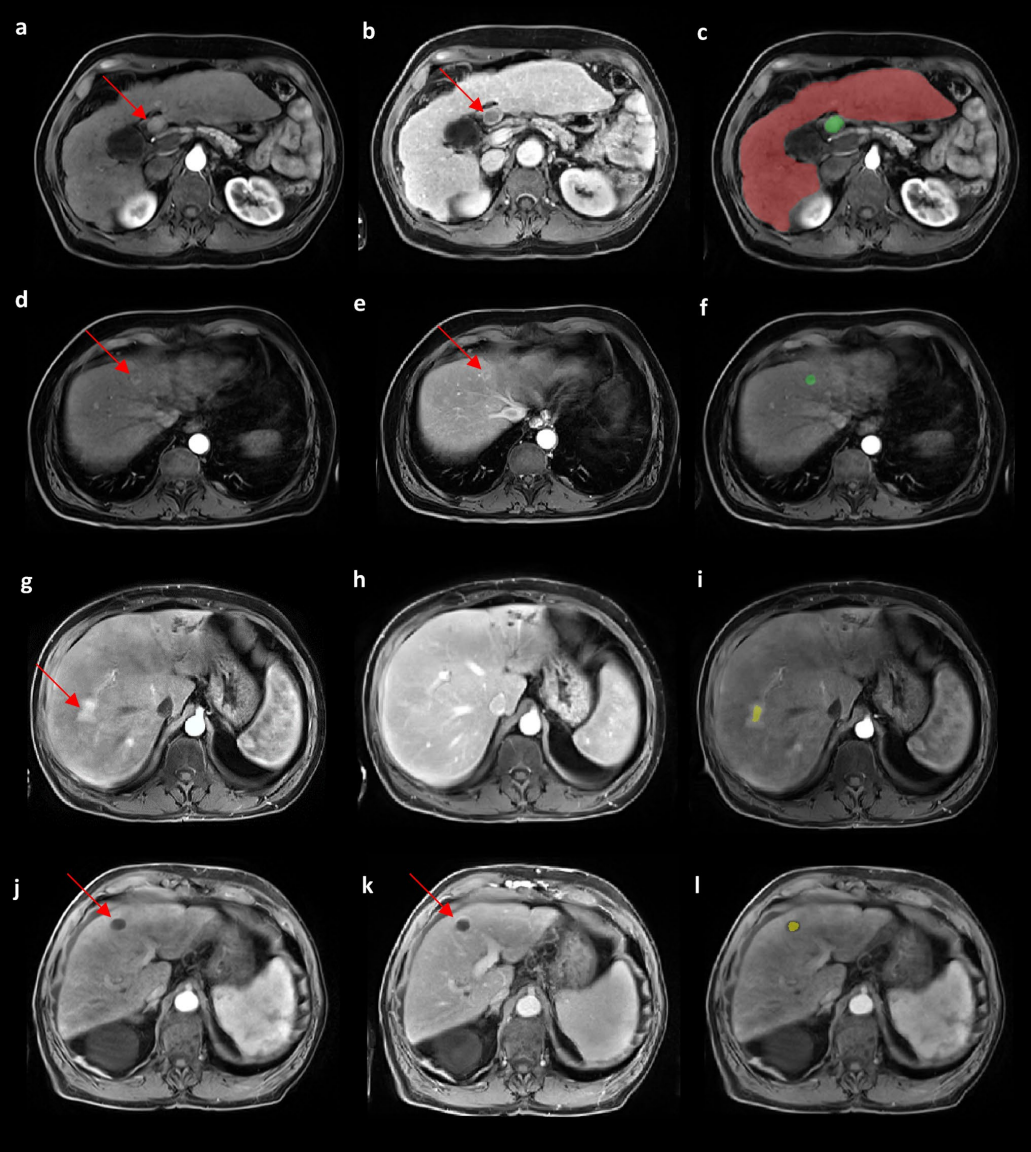
Examples of false positive and false negative predictions of the U-Net (Pre-training + Tversky) shown in the arterial phase and venous or delayed phase. In the first column on the left **a**, **d**, **g**, **j** are the arterial phases, in the second **b**, **e**, **h**, **k** are the corresponding venous or delayed phases and in the last **c**, **f**, **i**, **l** are the predictions and ground truth. The liver model predictions are highlighted in red (**c**), the tumor model predictions in yellow (**i**, **l**) and the tumor ground truth in green (**c**, **f**). **a–c** Example of false negative prediction due to liver segmentation error (under-segmentation), excluding the HCC lesion located in segment I (arrows). **d–f** Example of false negative prediction on a small (diameter of 12 mm, arrows) lesion with low contrast in arterial phase (**d**, arrow). **g–i** False positive prediction on a perfusion abnormality appearing hyper-intense on the arterial phase (**g**, arrow) and not visible on the other phases. **j–l** Example of false positive prediction on a simple cyst appearing hypo-intense on all phases (arrows). Full multiphasic visualizations are presented in Supplementary material 7. *HCC* hepatocellular carcinoma, *U-Net (Pre-training + Tversky)* U-Net pre-trained on LiTS and sequentially fine-tuned on LLD-MMRI and the HCC Surveillance test set

In the HCC Surveillance test set, a total of 6 HCC were not detected by the model among 16 HCC while in the HCC Pre-Ablation test set 21 HCC were missed among 104 HCC lesions. The missed lesions included 20 LR-5, 6 LR-4 and 1 LR-M (Fig. 6). Of them, 3 lesions were not included in the liver mask and couldn’t be seen by the model and 24 lesions seem to be missing due to low contrast enhancement in arterial phase and the small size of the lesion. A total of 69/233 HCC negative patients were predicted as HCC positive with a mean of 2.68 false positives per patient. These false positive predictions often overlapped hyper-intense regions on the arterial phase including fibrotic regions, perfusion abnormalities and MRI artefacts located near the liver margins. A few cases of T1WI hypo-intense regions such as cysts were detected as HCC (Fig. 6).

## Discussion

Our study demonstrates the feasibility of automatic HCC detection using deep learning model based on DCE MRI performed with extracellular contrast agent reaching 80% sensitivity at patient and lesion levels. To the best of our knowledge, our study is the first investigating automated HCC detection in extracellular gadolinium-enhanced MRI in a European population and the first to evaluate performance on a realistic, low HCC prevalence (~ 5%) test set, reflecting real-world screening conditions. At patient level, our fine-tuned U-Net (Pre-training + Tversky) model demonstrated a sensitivity of 83% and a specificity of 72%, detecting small HCC while preserving a low number of false positive predictions. These results outperform the standard screening guidelines with US reporting a sensitivity of 47% for small sized HCC in a large meta-analysis [2]. Our models achieved a low PPV (13.3% and 14.5%) but a very high NPV (98%), which is particularly important in a screening scenario to correctly exclude HCC negative patients, who represent the majority of the population at risk of HCC. At lesion level, 80% of the lesions in the HCC Pre-Ablation test set (*n* = 113) were detected with our fine-tuned U-Net (Pre-training + Tversky) model with a mean of 1 false positive per patient, significantly outperforming the baseline nnU-Net (*p* = 0.0009). In the analysis of the detection performance stratified by LIRADS scores, the LR-5 and LR-4 lesion stratification showed a significantly improved performance of our fine-tuned U-Net (Pre-training + Tversky) compared to the nnU-Net (*p* < 0.01 for HCC and LR-5 and *p* < 0.05 for LR-4).

We compared our study to similar published works and summarized the key aspects of each in Table 5. In our study, we achieved a HCC detection sensitivity of 80% with a mean of 1 false positive per patient across two test sets, which is in line with or slightly higher than previously reported results by Bousabarah et al. [6] (reporting 75% sensitivity using a U-Net model), though with a lower false positive rate and larger lesions (mean diameter ~ 29 mm vs. 24 mm in our study). That study lacked patient level specificity assessment, as no HCC negative patients were included, and liver disease etiology was not specified, despite its known impact on liver morphology and lesion conspicuity [10, 11]. Their data were also more homogenous compared to our study, as acquired with 1.5T and 3T Siemens MRI at a single academic center in the United States of America (USA). Zheng et al. [28] reported stronger lesion and patient level sensitivity (89% and 94%), using a method relying on a pattern-matching approach, searching for predefined imaging patterns in DCE T1WI and DWI and combined with a CNN to classify the matched regions. Differences in methodology, study population (mostly HBV/HCV-related chronic liver disease) and imaging homogeneity (3T Philips Ingenia only) make direct comparison challenging. Similarly, Luo et al. [8] obtained higher lesion level sensitivities (96–97%) on larger lesions (mean lesion size 58 mm), but large lesions are typically easy to depict by radiologists. Conversely, smaller sized lesions are more challenging, and it is where deep learning models could be beneficial. Moreover, the goal of HCC screening is to depict small-sized HCC, potentially curable rather than advanced tumors. This later study did not include HCC negative patients to assess specificity, which is crucial for evaluation in a more realistic scenario: indeed, prevalence of HCC in the screening population is typically between 1.5% and 8% according to chronic liver disease type [29]. Their data also originated from a homogenous Chinese population and imaging setup (3T MRI, 1–2 scanners). Lei et al. [30] used liver specific contrast MRI to detect small HCCs (mean 15.7 mm), reporting 88.2% sensitivity and 70% specificity on a near-balanced test set (63.8% HCC prevalence). Their data were also from a Chinese population with a narrow range of scanners and field strengths. In contrast, our study is the first to evaluate automated HCC detection in extracellular gadolinium-enhanced MRI in a European population and to evaluate the performance on a realistic low HCC prevalence (~ 5%) test set, in line with the actual surveillance conditions [29, 31]. We achieved 83% sensitivity and 72% specificity at the patient level, supporting the clinical relevance and generalizability of our model. In our HCC Surveillance test set, which is close to a realistic screening population with a low disease prevalence (~ 5%), the model achieved a PPV of 13.5% and a NPV of 98.8% at the patient level. Despite a relatively high number of false positives (69/233 HCC negative), the model showed excellent ability to correctly identify true negatives. This high NPV suggests that the model could be useful in reassuring the absence of disease. In the context of screening using abbreviated MRI, the model could be used to rule out HCC lesions and reduce radiologist workload [13].

Our cohort includes predominantly patients with alcohol-related chronic liver disease, may exhibit a hepatic architecture different (typically more advanced fibrosis) compared to Asian cohorts, dominated by hepatitis B, often without cirrhosis [10]. We also used heterogenous data which significantly differed in terms of demographics and clinical characteristics, including mean age, ethnicity, etiology of liver disease and Child-Pugh score (all p-values < 0.05). The data sets differed also with the acquisition equipment with MRI acquired from 3 manufacturers and 17 different scanners. Very few examples of 1.5T and no 1T were seen during training (96% of 3T) while the HCC Pre-Ablation test dataset was composed of 55% of 1T and 1.5T. Ten different MRI scanners from 3 manufacturers that were also not seen during training, suggesting the algorithm’s robustness and potential generalizability across different types of imaging (Supplementary material 1). These results show that our model achieves comparable diagnostic accuracy despite being tested in a larger, more imbalanced screening cohort with advanced underlying liver disease, underscoring its robustness.

Our study has several limitations, in particular the low number of lesions for training and testing our algorithm. Even though a large dataset (LLD-MMRI) was recently published publicly, there is still a need for more publicly available annotated data including small sized HCC lesions to train and evaluate deep learning models for screening purposes. The stratification evaluation is limited due to the low amount of LR-3 (10 lesions) and LR-4 (15 lesions) lesion examples present in our test sets. Another limitation comes from the liver segmentation model for extracting the liver from the DCE sequences, a few lesions located at the border of the liver were not covered by the liver mask prediction. Also, our model showed limitations to distinguishing other lesions or artefacts by predicting 69/233 HCC negative patients as HCC positives. The false positive predictions typically included perfusion abnormalities and artefacts located near the liver margins appearing hyper-intense on the arterial phase. These false positives lead to a patient level PPV of 13.5% which is largely lower than the 94–100% PPV reported in other studies. This difference is largely attributable to the markedly lower HCC prevalence in our HCC Surveillance test set (5%), whereas prior studies either excluded HCC negative patients or used datasets with artificially high prevalences (50–100%) and did not report prevalence-adjusted PPV/NPV. When compared on a 5% prevalence-adjusted basis in (Table 5), our results (13.5% PPV) are consistent with similar studies such as Lei et al. [30] (13.4% PPV). As PPV and NPV are strongly influenced by disease prevalence—with higher prevalence inflating PPV and lower prevalence inflating NPV [26]—the low prevalence in our test set explains the lower PPV and higher NPV relative to other reports. The false positive predictions may be reduced by incorporating T2WI and DWI to provide additional information about soft-tissue contrast, tissue cellularity and diffusion restriction, thus improving lesion characterization performance. As a future step, we will approach the task of lesion characterization in addition to that detection step.

**Table 5:** Comparison with literature

Study	Data origin	MRI brands	MRI scanners	Magnetic field strength	Sequence used	Contrast agent	Liver disease etiology	Model	Mean HCC size (mm)	Test set lesions	Test set Mean HCC diameter (mm)	Test set HCC pos/neg patients	HCC pre-valence	Patient level Se./Sp.	Patient level PPV/ NPV	Patient level adjusted PPV/NPV	Lesion level Se./Mean FP per patients	Lesion level PPV
Bousabarah et al. (2021) [6]	USA	1	–	1.5T 3T	DCE T1WI	Extra-cellular	–	DCNN	28.7 ± 13.7	~ 35	–	174	100%	–/–	–/–	–/–	75%/ 0.75	–
Zheng et al. (2021) [28]	KR	1	–	3T	DCE T1WI, DWI	Extra-cellular	HBV (70%) HCV (7%) Other (23%)	Pattern matching + CNN	< 20	38	–	32/32	50%	93.75%/ 100%	100%/ 94%	100%/ 99.7%	89%/–	85%
Luo et al. (2024) [8] (internal set)	CN	3	3	3T	DCE T1WI	Extra-cellular	–	Multi-phasic CNN	–	91	65.9 ± 41.1	76/–	100%	96%/–	94%/–	–/–	89%/–	88%
Luo et al. (2024) [8] (external set)	CN	3	3	3T	DCE T1WI	Extra-cellular	–	Multi-phasic CNN	–	62	32.6 ± 20.6	51/–	100%	97%/–	94%/–	–/–	94%/–	89%
Lei et al. (2025) [30]	CN	3	–	1.5T 3T	DCE T1WI	Hepato-specific	–	nnU-Net	15.7 ± 3.8	–	–	74/42	63.8%	88%/ 70%	–/–	13.4%/ 99.1%	–/–	–
Our work (HCC Surveillance)	EU	3	10	1T 1.5T 3T	DCE T1WI	Extra-cellular	Alcohol (54%) HCV (28%) HBV (16%)	U-Net (Pre-training + Tversky)	24.5 ± 13.8	21	23.5 ± 16.7	12/233	5%	83%/ 72%	13.3%/ 98.8%	13.5%/ 98.8%	81%/ 2.68	–
Our work (Pre-Ablation)	EU	3	14	1T 1.5T 3T	DCE T1WI	Extra-cellular	Alcohol (54%) HCV (28%) HBV (16%)	U-Net (Pre-training + Tversky)	24.5 ± 13.8	113	28 ± 18.7	67/–	100%	–/–	–/–	–/–	80%/ 0.89	–

*CN* China, *EU* Europe, *USA:*United States of America, *KR* South Korea, *DCE* dynamic contrast-enhanced, *HCV* hepatitis C virus, *HBV* hepatitis B virus, *HCC* hepatocellular carcinoma, *DCNN* deep convolutional neural network, *CNN* convolutional neural network, *PPV* positive predictive value, *NPV* negative predictive value, *Se* Sensitivity, *Sp* Specificity, – not reported, “: same as above

## Conclusion

Our results demonstrate the potential of deep learning algorithms for liver lesion detection in patients at risk of HCC, with performance achieved under prevalence conditions closely matching those of real-world clinical screening. A high sensitivity at lesion and patient level as well as high NPV were achieved with limited data and could greatly help professionals in their clinical daily routine to rule out for HCC lesion and reduce reading time, which could be beneficial in a screening perspective.

## Supplementary Information

Below is the link to the electronic supplementary material.

Supplementary Material

## Data Availability

A portion of the data supporting the findings of this study will be made publicly available in an open-access repository upon publication.

## Abbreviations

MRI: Magnetic resonance imaging
HCC: Hepatocellular carcinoma
LI-RADS: Liver imaging reporting and data system
US: Ultrasound
WI: Weighted imaging
CT: Computed tomography
DCE: Dynamic contrast-enhanced
