# Supplementary material for: Deep learning for automatic detection of hepatocellular carcinoma in dynamic contrast-enhanced MRI
Source: Abdom Radiol (NY). 2025 Nov 11;51(6):2843–56. doi: 10.1007/s00261-025-05249-4 (PMC13109151; doi:10.1007/s00261-025-05249-4)
Supplement: Supplementary file 1 — Supplementary Material [file 261_2025_5249_MOESM1_ESM.docx]

**Supplementary materials**

**Supplement material 1**: MRI Acquisition

**Acquisition protocols:**

The protocol for image acquisition in the HCC Surveillance cohort included a breath-hold axial T1WI using a fat-suppressed three-dimensional spoiled gradient-echo sequence, volumetric interpolated (VIBE) obtained before and after contrast injection. An intravenous injection of 0.1 mmol/kg of gadoterate meglumine (Dotarem; Guerbet) at a rate of 3 ml/s was then performed, followed by a bolus of 20 ml of saline solution at a rate of 2 ml/s. Triple arterial phase images were acquired after a delay of 20 seconds with a Caipirinha (controlled aliasing in parallel imaging results in higher acceleration) Dixon TWIST (time-resolved angiography with stochastic trajectories) volume interpolated breath-hold examination sequence (VIBE). Portal venous, equilibrium and delayed phases were acquired, at 70 sec, 3 min and 5 min, respectively [13].

The protocol for image acquisition in the HCC Pre-Ablation cohort included gadoxetic-acid-enhanced dynamic T1-weighted images with a fat-suppressed 3D spoiled gradient-echo sequence, with volumetric interpolated breath-hold examination (VIBE; Siemens Healthcare, Erlangen, Germany) following a bolus injection (0.2 mL/kg) of gadoteric acid (Dotarem; Guerbet, Villepinte, France), before contrast administration and in the arterial phase (30s after contrast administration), venous phase (70s after contrast administration), and delayed phase (3min after contrast administration) [14].

**MRI Machines:**

The HCC Surveillance cohort included mostly 3T MRI with 96% of 3T and only 3% of 1.5T in each sub-dataset. In the HCC Pre-Ablation test set, 3% of the MRI were 1T, 52% of 1.5T and 45% of 3T. The training/validation and test set from the HCC Surveillance cohort included 100% and 98% of their data from Siemens equipment. The HCC Pre-Ablation cohort included 80% of data acquired with Siemens, 13% from Philips and 5% from GE equipment. The MRI scanners used to acquire data were substantially different between the 2 cohorts. The HCC Surveillance training/validation set included 6 different scanners and the test set 10 scanners. The HCC Pre-Ablation included MRI from 14 scanners. The voxel spacing was slightly higher in the HCC-Pre-Ablation set (3.16±0.78) compared with the HCC Surveillance set (3.06±0.23 in the training/validation set and 3.09±0.31 in the test set). Similarly, the slice thickness was greater in the HCC Pre-Ablation set (3.58±0.81) compared to the HCC Surveillance set (3.06±0.24 in the training/validation set and 3.11±0.29 in the test set).

**Table S1:** MRI Machine and Imaging Parameters Comparison

|  |  | **HCC  Surveillance** | | **HCC  Pre-Ablation** |
| --- | --- | --- | --- | --- |
|  |  | Training/ Validation | Test | Test |
| Field strength | 1T | 0 (0%) | 0 (0%) | 2 (3%) |
|  | 1.5T | 2 (3%) | 8 (3%) | 35 (52%) |
|  | 3T | 49 (96%) | 237 (96%) | 30 (45%) |
| Manufacturer | GE | 0 (0%) | 1 (0%) | 4 (5%) |
|  | Philips | 0 (0%) | 2 (0%) | 9 (13%) |
|  | Siemens | 51 (100%) | 242 (98%) | 54 (80%) |
| Scanner | Aera | 1 (1%) | 6 (2%) | 12 (17%) |
|  | Avanto | 0 (0%) | 1 (0%) | 4 (5%) |
|  | DISCOVERY MR750 | 0 (0%) | 0 (0%) | 1 (1%) |
|  | Espree | 0 (0%) | 0 (0%) | 4 (5%) |
|  | Ingenia | 0 (0%) | 2 (0%) | 4 (5%) |
|  | Integrity Medical Image Importer | 1 (1%) | 1 (0%) | 4 (5%) |
|  | Intera | 0 (0%) | 0 (0%) | 1 (1%) |
|  | MAGNETOM Vida | 0 (0%) | 1 (0%) | 0 (0%) |
|  | Optima MR450w | 0 (0%) | 0 (0%) | 3 (4%) |
|  | Panorama HFO | 0 (0%) | 0 (0%) | 1 (1%) |
|  | Prisma fit | 1 (1%) | 4 (1%) | 0 (0%) |
|  | Skyra | 40 (78%) | 201 (82%) | 17 (25%) |
|  | Skyra fit | 5 (9%) | 13 (5%) | 0 (0%) |
|  | Symphony | 0 (0%) | 0 (0%) | 7 (10%) |
|  | SymphonyTim | 0 (0%) | 0 (0%) | 1 (1%) |
|  | TrioTim | 0 (0%) | 1 (0%) | 3 (4%) |
|  | Verio | 3 (5%) | 15 (6%) | 5 (7%) |
| Image dimensions (mean ± std) | X | 195±47 | 184±37 | 207±77 |
|  | Y | 162±42 | 155±26 | 182±67 |
|  | Z | 54±9 | 54±11 | 53±13 |
| Voxel spacing (mean ± std) | X | 1.19±0.14 | 1.20±0.12 | 1.13±0.33 |
|  | Y | 1.19±0.14 | 1.20±0.12 | 1.13±0.33 |
|  | Z | 3.06±0.23 | 3.09±0.31 | 3.16±0.78 |
| Slice thickness (mean ± std) | | 3.06±0.24 | 3.11±0.29 | 3.58±0.81 |

**Supplementary material 2:** Neural networks, pre-processing and post-processing

***Attention gate***


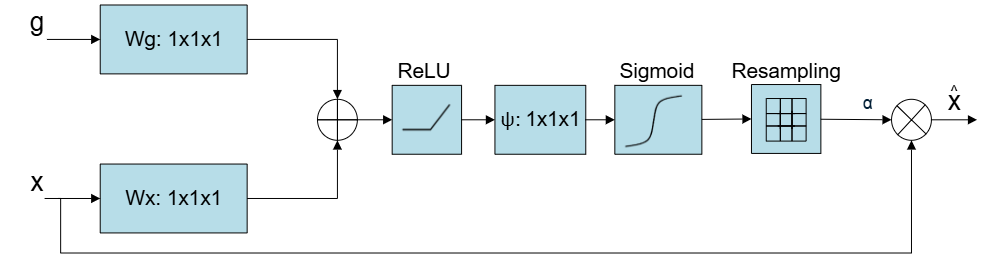


**Fig. S1** Schematic of the additive attention gate (AG) used in the Attention U-Net architecture [23]. Input features (x) are scaled with attention coefficients (α) computed in AG spatial regions are selected by analyzing both activations and contextual information provided by the gating signal (g) which is collected from a coarser scale. Grid resampling of attention coefficients is done using trilinear interpolation. Figure adapted from Oktay et al. [23].

***Pre-processing***

Bias field correction was applied to all T1WI DCE images to remove field inhomogeneities, using SimpleITK v2.4.0 [18]. A trained nnU-Net [19] for liver segmentation was then used to generate the liver mask of each phase of the DCE T1WI phases. These masks were then used to perform a first rigid + non-rigid pair-wise co-registration of each phase to the venous phase using the Elastix Python library version 5.0.1 [20]. A second group-wise registration [21] was then applied to fine-tune the alignment of the liver and the lesions. The liver from the resulting 4D image was extracted with the liver mask generated by the nnU-Net. The voxel intensities of each phase of the 4D images were independently scaled from the 5^th^ percentile to the 100^th^ percentile to 0 and 1. The voxel dimensions were resampled with the lowest voxel dimension found of 0.59 x 0.59 x 3.0 mm^3^.

***Neural Networks***

**nnU-Net:** The network architecture was the 3D nnU-Net with [32, 64, 128, 256, 320, 320] channels. The data were pre-processed by applying Z-score normalization and resampled the voxel spacing to 3x1.1875x1.1875. The loss function was the DC and CE loss (a combination of the robust cross-entropy loss and the soft Dice loss). The learning rate was initialized to 0.01 and decreased with a polynomial learning rate scheduler. The optimizer was the SGD optimizer with a momentum of 0.99 and a weight decay of 3x10^-5^. The number of epochs was set to 250. The batch size was set to 2 and the patch size was set to 192x160x56. The 4D images were automatically handled as multi-modal data for each phase of the DCE MRI.

**U-Net (Tversk):** The first model was trained from scratch with all the available lesion annotations to create a model sensitive to all types of lesions. To do so the input patch size was set to a small size of 64x64x16. The architecture was the Attention U-Net [23] implemented in the MONAI library. The model was initialized with [32, 64, 128, 256, 320, 320] channels and [2, 2, 2, 2, 1] strides. The model was trained with a batch size of 2 for 3000 epochs. Each batch consisted of five random crops with a ratio of foreground and background set to 1:1. For each patient the tumors were defined as foreground and the healthy regions of the liver as background. The Adam optimizer was used with a learning rate initialized at 1x10^-4^. The Tversky loss function was defined with α=1.0 and β=0.9 to penalize false positive predictions less, and false negatives more, creating a sensitive model. Polynomial learning rate scheduling was also used to obtain a more stable convergence. Data augmentation was done on the fly with MONAI transform functions RandAdjustContrast (P=0.15) with γ range of [1, 1.5] and RandFlip along x and y axis (P=0.5). The segmentation results were obtained using a sigmoid activation function followed by a threshold of 0.5.

**U-Net (Pre-trained + Tversky):** The second model was tailored for the detection of HCC lesions. All the lesion annotations were used but a 10 times greater penalization was set on HCC lesions to teach the model to recognize only HCC. The patch size was increased to 128x128x16 to provide more context to the model and the Tversky loss was used with α=0.9 and β=0.1 to reduce false positive detections. The architecture of the model was again the Attention U-Net from the MONAI library. The model was pre-trained from scratch on the LiTS dataset [16] (489 patients), fine-tuned on LLD-MMRI dataset [17] (131 patients) and then further fine-tuned with our internal dataset. That pre-training was used to progressively prepare the model to recognize HCC lesions with similar tasks. The pre-training task on LiTS was the detection of various liver lesions on Computed Tomography (CT) imaging. The training configuration was left the same as for the previous experiment but for data augmentation, RandFlip was extended along x, y and z axis (P=0.5). After the initial pre-training phase, the model was fine-tuned to detect the lesions in DCE MRI using the LLD-MMRI dataset. Finally, it was further fine-tuned with our Training and Validation Set. The maximum number of epochs was set to 6000 for pre-training on LiTS and LLD-MMRI and to 3000 epochs for the final finetuning.

***Post-processing***

The output probability maps were obtained with sliding window inference from the MONAI library with an overlap of (0.5, 0.5, 0.5) for the x, y, z directions. The Gaussian mode was employed to assign more weight to the center of each patch with a sigma scale of (0.25, 0.25, 0.25). Test time augmentation with 5 random flips along x and y axis was used to improve prediction reliability and were combined by mean averaging. Additionally, local temperature scaling was used to calibrate the output predictions [24].

Recursive Gaussian blurring (sigma=1) was applied before thresholding the probability map to reduce noise and artefacts. Erosion and dilation were used after thresholding to remove remaining artefacts and improve the separation of distinct components within the prediction map. Predictions with a diameter smaller than 10mm were removed. Anatomical masks were generated using TotalSegmentator [25] and were employed as a post-processing step to remove predictions on the vena cava, the portal vein and the kidneys when part of them were wrongly integrated into the liver mask. The predictions for the test sets were obtained using a mean ensembling method, averaging the predictions from the 5-folds cross-validation models.

**Supplementary material 3:** Statistical methods

For FROC curves calculation, we computed the sensitivity and the mean number of false positives per patient for several probability map thresholds. The metrics were computed on a lesion-wise basis, calculated across the entire dataset without considering patient-specific segmentation, ensuring an independent assessment of each lesion, regardless of the individual patient context. The true positive (TP) detection of a lesion was defined as a non-zero overlap between the predicted region and the ground truth lesion. False positive (FP) predictions were defined as any prediction not overlapping with a ground truth lesion. False negative (FN) were defined as any ground truth lesion for which no overlapping region was predicted.

For permutation tests, the TP, FP, FN metrics per patient used to calculate the FROC curves were randomly permuted 1000 times to test the null hypothesis that the observed differences in AUC values between models occurred by chance.

**Supplementary material 4:** Validation set performance stratification

**Table S2**: FROC Curves AUC

| Validation set | | | | | | |
| --- | --- | --- | --- | --- | --- | --- |
|  | All lesions (n=113) | HCC  (n=83) | LR-5  (n=73) | LR-4  (n=22) | LR-3  (n=18) |  |
| nnU-Net | 0.53 | **0.61** | **0.60** | 0.44 | 0.27 |  |
| U-Net (Tversky) | 0.27 | 0.33 | 0.33 | 0.18 | 0.08 |  |
| U-Net (Pre-training + Tversky) | **0.56** | 0.59 | 0.59 | **0.59** | **0.41** |  |

**Supplementary Materials 5: Results with different thresholds on IoU**

**Table S3**. Detection metrics with different IoU thresholds

|  | IoU > 0 | IoU > 0.1 | IoU > 0.2 | Mean FP per patient |
| --- | --- | --- | --- | --- |
| **HCC Surveillance Test Set** |  |  |  |  |
| nnU-Net | 56% | 56% | 50% | **0.27** |
| U-Net (Tversky) | 62% | 62% | 62% | 2.5 |
| U-Net (Pre-training + Tversky) | **81%** | **75%** | **69%** | 2.68 |
|  |  |  |  |  |
| **HCC Pre-Ablation Test Set** |  |  |  |  |
| nnU-Net | 63% | 56% | 50% | **0.45** |
| U-Net (Tversky) | 59% | 57% | 57% | 0.92 |
| U-Net (Pre-training + Tversky) | **80%** | **74%** | **71%** | 0.89 |

**Supplementary material 6:** Full multiphasic example for true positive and false negative predictions


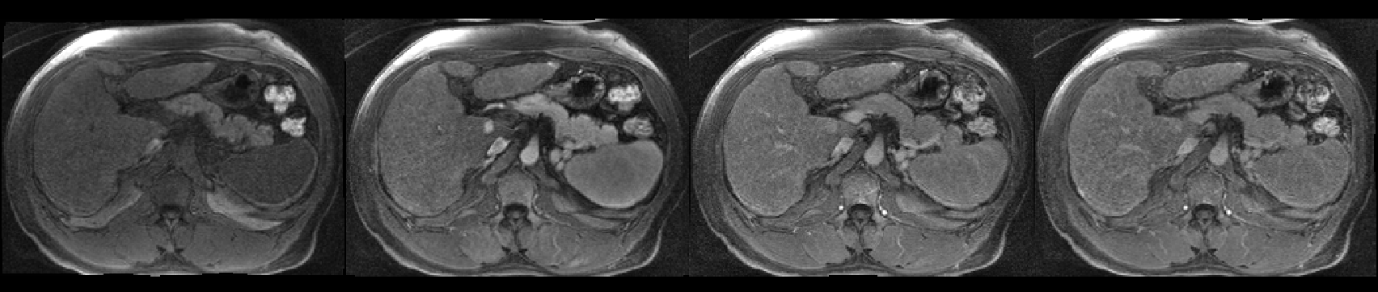

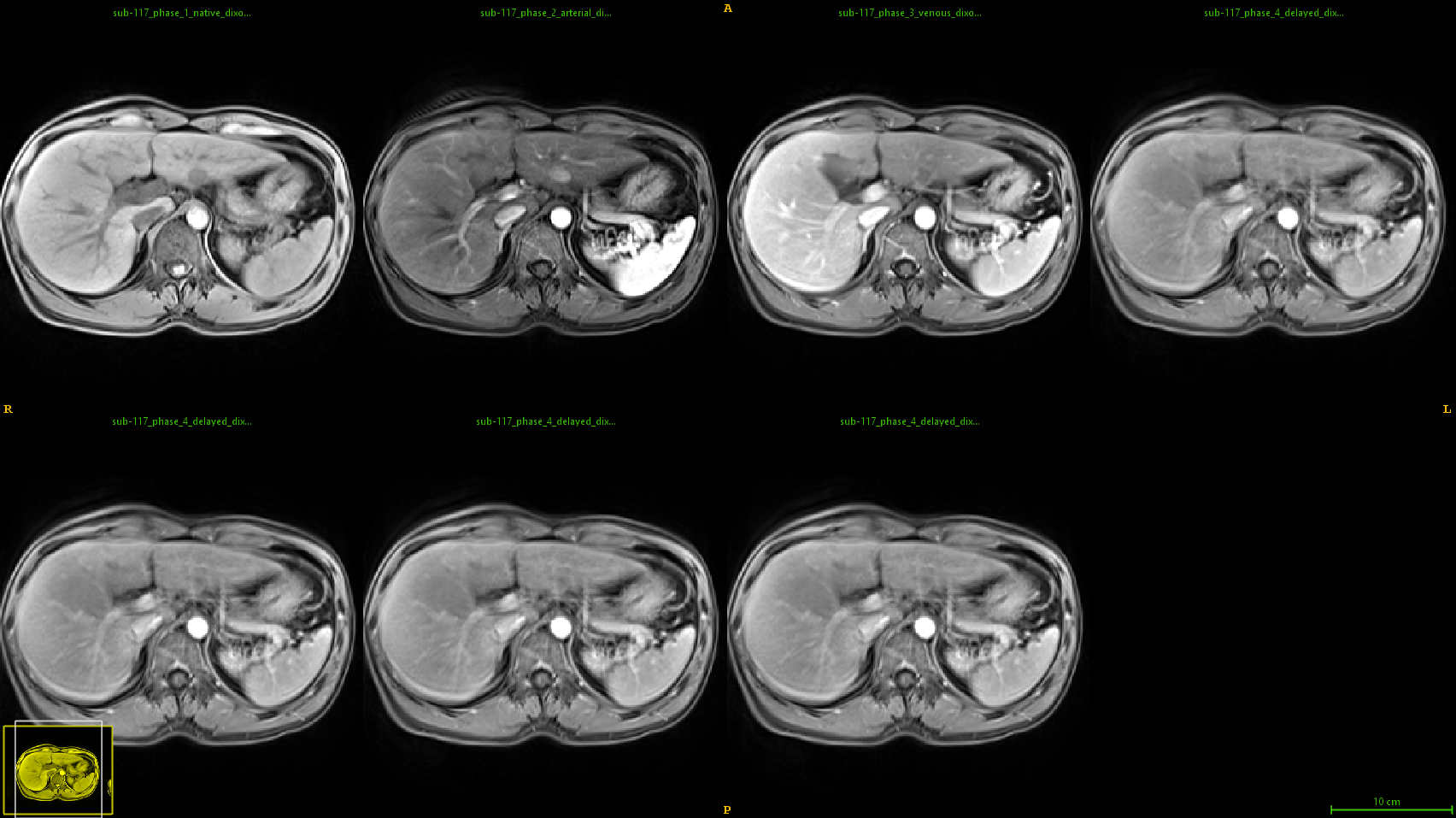


**a**

**e**

**b**

**c**

**d**

**f**

**g**

**h**

**Fig. S2** Multiphasic image of cases with true positive and true negative predictions of the U-Net (Pre-training + Tversky) presented in **Figure 5**. The images are presented in order from left to right with the native (pre-contrast **a** and **e**), arterial (**b** and **f**), venous (**c** and **g**) and delayed phases (**d** and **h**). **a-d)** Example of an HCC lesion (arrows) with hyperenhancement in arterial phase (**b**) and washout in venous and delayed phases (**c** and **d**). **e-h)** Example of a non-HCC lesion (dysplastic nodule) with arterial phase hyperenhancement (**f, arrow**), without washout nor pseudo capsule on venous and delayed phases (**g** and **h**). Abbreviations: HCC: hepatocellular carcinoma. U-Net (Pre-training + Tversky). Abbreviations: U-Net (Pre-training + Tversky): U-Net pre-trained on LiTS and sequentially fine-tuned on LLD-MMRI and the HCC Surveillance test set.

**Supplementary material 7:** Full multiphasic examples of false negative and false positive cases


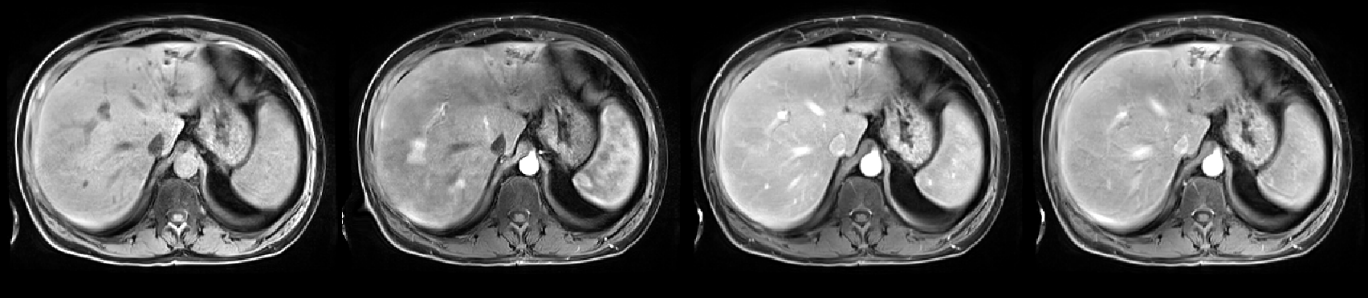

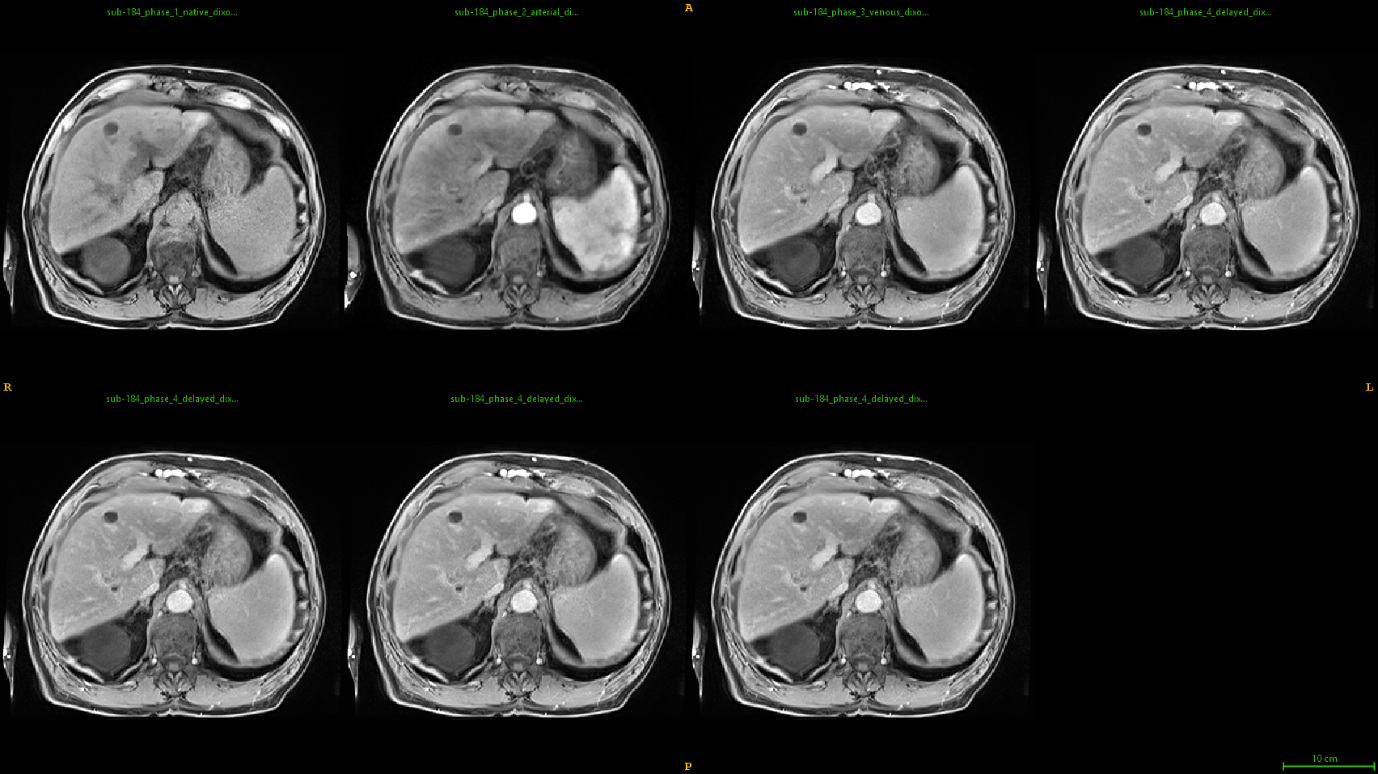

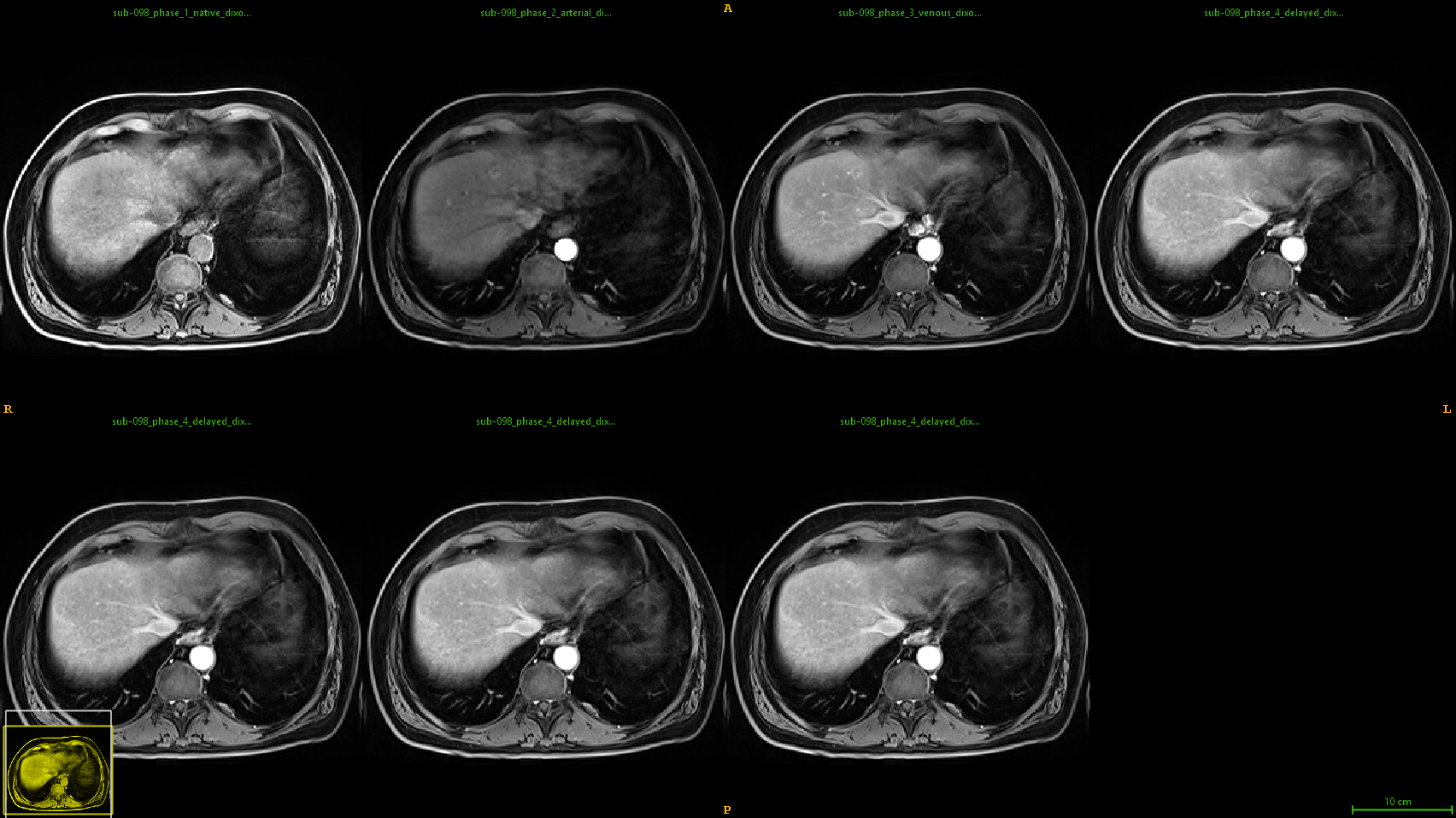

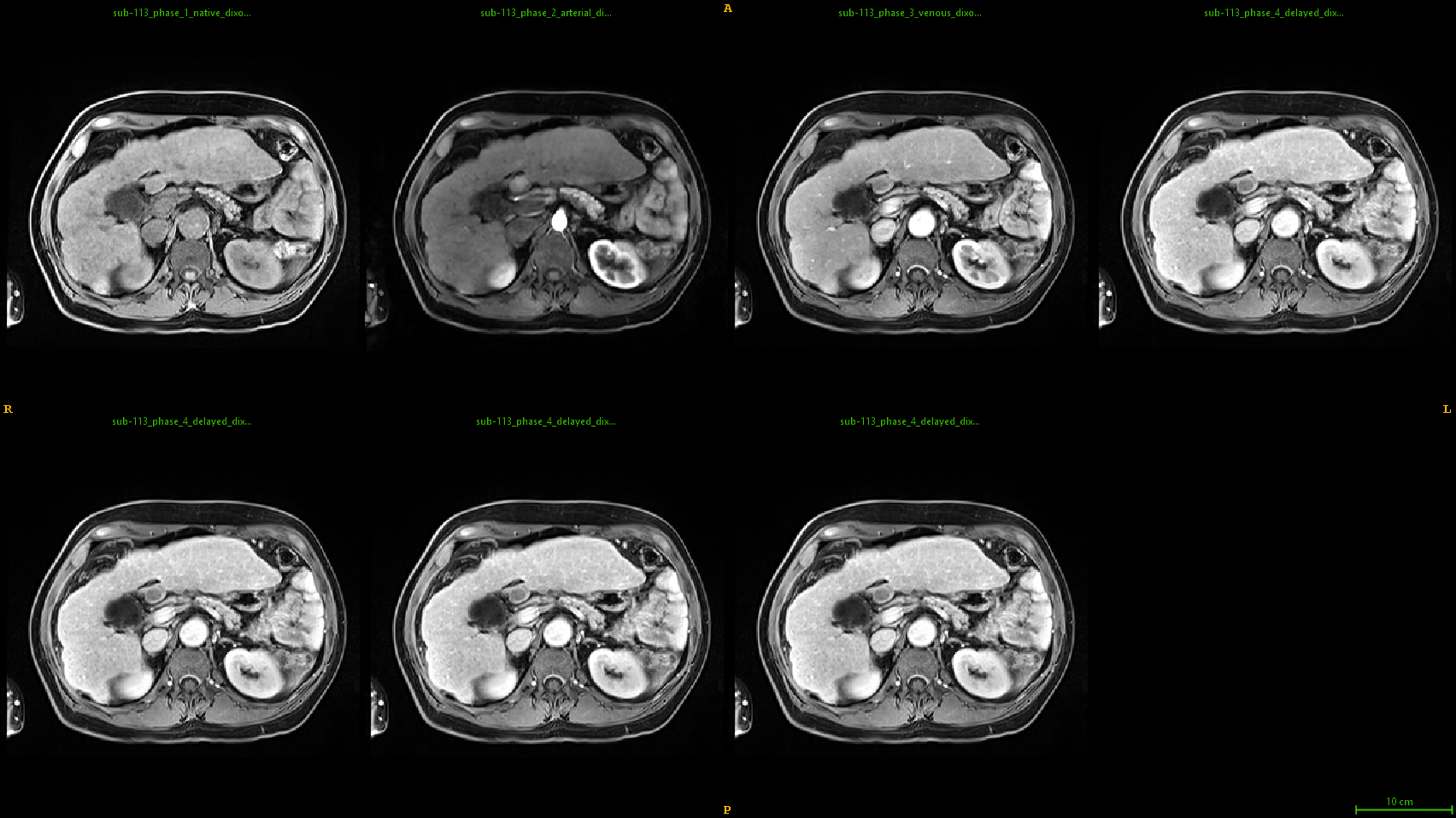


**a**

**i**

**e**

**m**

**b**

**j**

**f**

**n**

**c**

**k**

**g**

**o**

**d**

**l**

**h**

**p**

**Fig. S3** Multiphasic image of cases with false positive and false negative predictions of the U-Net (Pre-training + Tversky) presented in **Figure 6**. The images are presented in order from left to right with the native (pre-contrast **a**, **e**, **i**, **m**), arterial (**b**, **f**, **j**, **n**), venous (**c**, **g**, **k**, **o**) and delayed phases (**d**, **h**, **l**, **p**). **a-d)** Example of a HCC lesion located in segment I (arrows). **e-h)** Example of a small lesion (diameter of 12mm) with low contrast in arterial phase (**f**). **i-l)** Example of a perfusion abnormality appearing hyper-intense on the arterial phase (**j, arrow**) and not visible on the other phases. **m-p)** Example of a simple cyst appearing hypo-intense on all phases (arrows). Abbreviations: HCC: hepatocellular carcinoma, wi: weighted imaging, U-Net (Pre-training + Tversky): U-Net pre-trained on LiTS and sequentially fine-tuned on LLD-MMRI and the HCC Surveillance test set.
